# Impaired rich-club connectivity in childhood absence epilepsy

**DOI:** 10.3389/fneur.2023.1135305

**Published:** 2023-05-11

**Authors:** Yadong Yu, Mengdi Qiu, Wenwei Zou, Ying Zhao, Yan Tang, Jisha Tian, Xiaoyu Chen, Wenchao Qiu

**Affiliations:** ^1^Department of Neurology, Lianshui County People's Hospital, Huai'an, China; ^2^Department of Neurology, The Fifth People's Hospital of Huai'an, Huai'an, China; ^3^Department of Neurology, The Affiliated Huai'an Hospital of Xuzhou Medical University, Huai'an, China; ^4^Department of Radiology, The Affiliated Huai'an Hospital of Xuzhou Medical University, Huai'an, China

**Keywords:** childhood absence epilepsy, diffusion tensor imaging, probabilistic tractography, graph theory, rich-club

## Abstract

**Introduction:**

Childhood absence epilepsy (CAE) is a well-known pediatric epilepsy syndrome. Recent evidence has shown the presence of a disrupted structural brain network in CAE. However, little is known about the rich-club topology. This study aimed to explore the rich-club alterations in CAE and their association with clinical characteristics.

**Methods:**

Diffusion tensor imaging (DTI) datasets were acquired in a sample of 30 CAE patients and 31 healthy controls. A structural network was derived from DTI data for each participant using probabilistic tractography. Then, the rich-club organization was examined, and the network connections were divided into rich-club connections, feeder connections, and local connections.

**Results:**

Our results confirmed a less dense whole-brain structural network in CAE with lower network strength and global efficiency. In addition, the optimal organization of small-worldness was also damaged. A small number of highly connected and central brain regions were identified to form the rich-club organization in both patients and controls. However, patients exhibited a significantly reduced rich-club connectivity, while the other class of feeder and local connections was relatively spared. Moreover, the lower levels of rich-club connectivity strength were statistically correlated with disease duration.

**Discussion:**

Our reports suggest that CAE is characterized by abnormal connectivity concentrated to rich-club organizations and might contribute to understanding the pathophysiological mechanism of CAE.

## 1. Introduction

The human brain is a network. The brain function is not solely attributable to the properties of individual regions or individual connections but rather emerges from the effective communication among regions linked within a complex network of white matter pathways, known as the human connectome (1). Conversely, brain dysfunction may also result from abnormal wiring of the brain's network.

Childhood absence epilepsy (CAE) is defined by the presence of multiple absence seizures in previously healthy developing children without a particular history of neurologic diseases. Typical seizures usually begin between 4 and 10 years of age (2). In the ILAE definition, the typical absence seizure is characterized by sudden cessation of ongoing activities with or without automatisms, usually lasting 30–60 s. The classic electroencephalogram (EEG) shows a generalized symmetrical and synchronous spike-wave discharges (GSWDs) pattern at 3 Hz with normal background activity. It affects 10–17% of all children with epilepsy, making it the most common form of generalized epilepsy syndrome in school-aged children (3). Although labeled “benign,” children with CAE may experience many times daily, including both clinical and subclinical seizures, which can impair memory processing and sustained attention. There is a strong association between CAE and significant cognitive deficits, behavioral disorders, and psychosocial difficulties (4–7). Given the “not so benign nature” of CAE, more attention is needed for this common childhood epileptic syndrome.

Based on some modern research studies (8, 9), the ILAE has recommended epilepsy as a network disease in their latest guidelines (10). Although the absence seizure is classified as a generalized onset, the generalized seizure is traditionally considered as the entire brain may be homogeneously embraced. There is emerging evidence supporting that generalized seizures are not truly generalized but rather rising in some regions and rapidly engaging bilaterally distributed networks containing the original regions (2).

The densely interconnected and central brain regions, so-called hub nodes, play a key role in sustaining efficient communication across the global brain network (11–13). In the cases of possible disruptions of information integration in epilepsy, the hub nodes may be of particular interest because hub regions have been suggested to be more vulnerable than non-hub regions in many brain disorders (14, 15). In the human brain, network hubs show higher connectivity with each other, beyond what would be expected based on their degrees, giving rise to the presence of the so-called “rich-club” phenomenon. The rich-club topology plays a pivotal role in the integrative capacity among regions of the whole-brain network (16). In our previous study, 12 hub regions and disrupted global integration of information have been detected in CAE (17). However, it is unclear whether these patients got disturbed wiring of rich-club topology and whether the global disruptions were concentrated to the rich-club connections.

In this study, diffusion tensor imaging (DTI) scans were acquired from 30 patients diagnosed with CAE and 31 healthy controls (HCs), and probabilistic tractography was employed to reconstruct the structural network. Based on the graph theory, the rich-club organizations of the structural brain network were compared between CAE and HCs. We hypothesized that the disturbed rich-club topology may contribute to the pathophysiology of CAE.

## 2. Materials and methods

### 2.1. Participants

A total of 30 clinically definite CAE patients and 31 healthy controls were recruited from the Department of Neurology at Nanjing Brain Hospital Affiliated with Nanjing Medical University and the Department of Neurology at the Affiliated Huai'an Hospital of Xuzhou Medical University. The CAE was diagnosed according to the guideline from the International League Against Epilepsy (ILAE) (2). This study was performed according to the Declaration of Helsinki and approved by the Ethics Committee of the Nanjing Brain Hospital Affiliated with Nanjing Medical University and the Affiliated Huai'an Hospital of Xuzhou Medical University. Informed consent was signed by all participants or their legal guardians prior to participation. All subjects are right-handed. The populations did not receive a significant difference in terms of age (CAE, mean and standard deviation of 8.30 ± 1.66; HCs, mean and standard deviation of 8.52 ± 1.61; *t*-test *p*-value = 0.61) or gender (16 female participants and 14 male participants for CAE; 18 female participants and 13 male participants for HCs; chi-squared test *p*-value = 0.71). More details of the demographic and clinical characteristics of each group are summarized in Table 1. The inclusion criteria for patient recruitment were as follows: (1) routine video-EEG showing bilateral, symmetrical spike-waves at approximate 3 Hz on normal background activity accompanied by clinical absence seizure; (2) normal neurologic development and no other seizure types; and (3) normal findings in the 3.0 T MRI. Among the 30 patients, either monotherapy or polytherapy of anti-seizure medications (ASMs) were accepted (for details, please see Supplementary Table S1).

**Table 1:** Baseline characteristics of participants.

	**CAE (*n* = 30)**	**HCs (*n* = 31)**	***P*-value**
Age (years, mean ± SD)	8.30 ± 1.66	8.52 ± 1.61	0.61
Gender (F/M)	16/14	18/13	0.71
Disease duration (months, mean ± SD)	9.33 ± 5.93	NA	-
Seizure frequency (times/day, mean ± SD)	8.27 ± 5.55	NA	-

M, male; F, female; CAE, childhood absence epilepsy; HCs, healthy controls; SD, standard deviation; NA, not applicable.

### 2.2. MRI acquisition and processing

The MR acquisition has been described previously (17). In brief, neuroimaging data were acquired on a Siemens 3.0 T scanner (Erlangen, Germany) equipped with a 12-channel head coil for sensitivity-encoding parallel imaging. Each subject underwent MRI scans as their head was restrained by foam pads to minimize head motion. Diffusion-weighted data were acquired using a single-shot echo planar imaging sequence (TR = 6600 ms, TE = 93 ms, acquisition matrix = 128 × 128, FOV = 240 × 240 mm^2^, 45 axial slices, slice thickness = 3 mm, and inter-slice gap = 0 mm). The diffusion weighting was isotropically distributed in 30 non-collinear directions (b = 1000 m^2^/s) with one additional acquisition without diffusion weighting (b = 0m^2^/s). High-resolution T1-weighted MRI was also obtained using a 3D rapid acquisition gradient echo sequence with the following scanning parameters: TR = 1900 ms, TE = 2.48 ms, acquisition matrix = 512 × 512, FOV = 250 × 250 mm^2^, 176 sagittal slices, slice thickness = 1 mm, and flip angle = 9°.

Using Diffusion Toolbox in FSL (FMRIB Software Library, http://fsl.fmrib.ox.ac.uk/fsl) (18), the DTI data were pre-processed to correct for eddy currents and head motion (19, 20). After fitting the diffusion tensor to the corrected images (21), the fractional anisotropy (FA) map was produced using the Diffusion Toolbox in FSL. In addition, the skull and surrounding soft tissues were removed using the FSL Brain Extraction Toolbox (BET) (22).

### 2.3. Construction of structural brain networks

**Figure 1:**
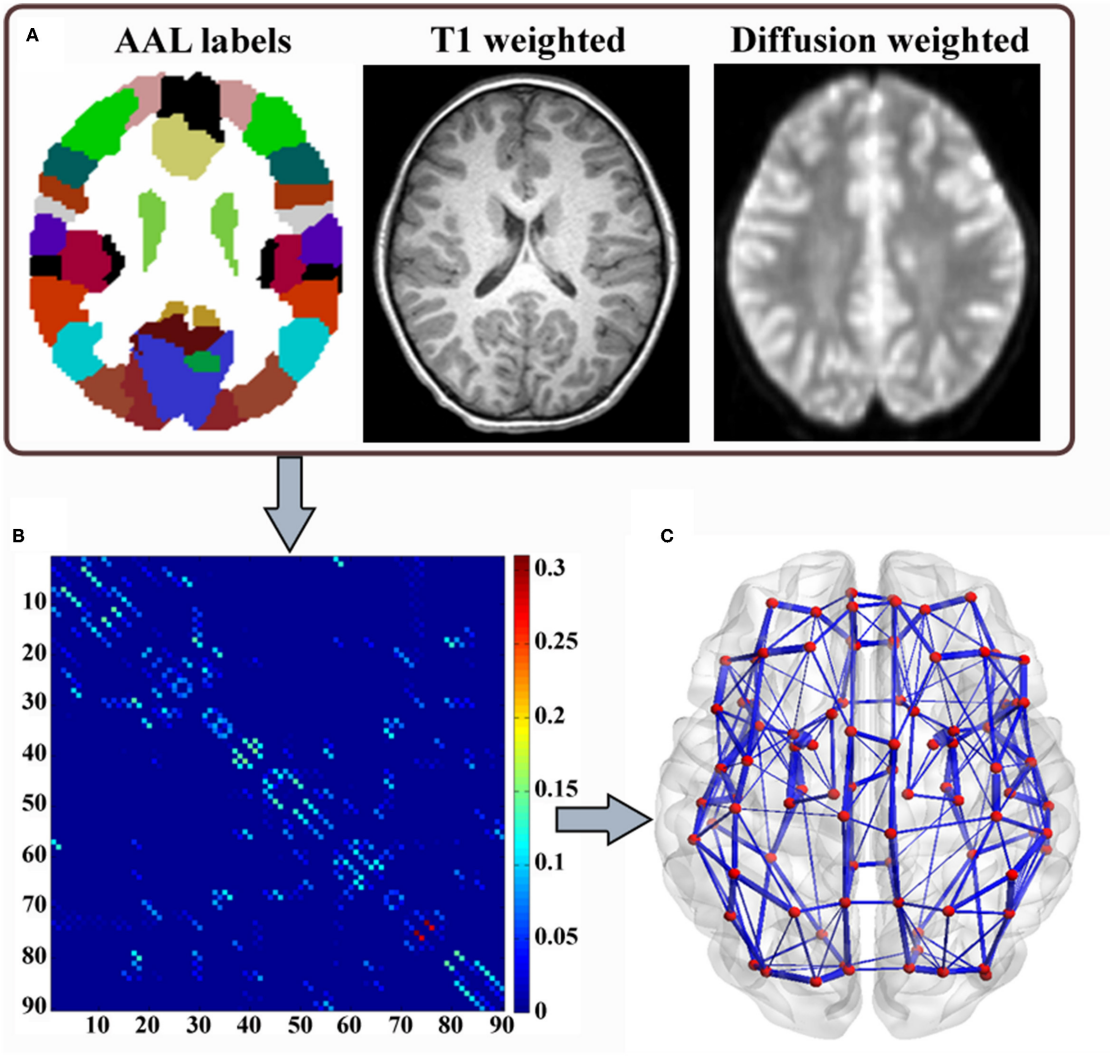
Flowchart for the construction of brain network. **(A)** Automated anatomical labeling (AAL) atlas, high-resolution T1-weighted MRI, and diffusion tensor images. **(B)** The connectivity matrix is generated using the probabilistic tractography algorithm after the brain is parcellated into a number of segregated regions and normalized to standard MNI space. **(C)** A weighted network is rendered by a 3D visualization model in BrainNet Viewer (BrainNet Viewer 1.53, Beijing Normal University, http://www.nitrc.org/projects/bnv/). The edges are encoded with their connection weights at the threshold of 0.01.

In graph theory, a network consists of a set of nodes connected by edges and can be mathematically expressed as a graph: *G* = (*V, E*), with *V* indicating a finite set of nodes and E indicating the set of edges between them (23). See Figure 1 for an overview of the analytical flowchart. This is the same as our previous study (17).

### 2.4. Network node definition

The procedure to define network nodes was completed following our previous study (17). In brief, the automated anatomic labeling (AAL) atlas was employed to generate 90 cortical and sub-cortical regions in diffusion native space (45 for each hemisphere, see Supplementary Table S2) (24). First, T1 images were non-linearly converted to the ICBM152 T1 template in the MNI space. Then, the AAL atlas was warped to the DTI native space by using the inverse transformation matrix. Of note, a nearest-neighbor interpolation method was employed in this step to preserve discrete labeling values. Finally, 90 AAL regions were executed and served as network nodes in the subsequent topological analyses.

### 2.5. Tractography-based structural connections

To define the edges of the brain network, probabilistic tractography was carried out using the FSL Diffusion Toolbox (FDT) (25). The bedpostx was run to estimate distributions on diffusion parameters at each voxel, and the probtrackx tool was used to conduct probabilistic tracking. From each voxel in the seed region, 5,000 samplings from the distributions of voxel-wise principal diffusion directions were repetitively performed. For each time, we can get a probabilistic streamline. After that, the connectivity probability from the seed region to each of the other 89 regions was calculated. As a result, a 90 × 90 network matrix *N* for each subject was generated. In the network matrix *N*, the element *Nij* means the connectivity probability from *i* to *j*. However, considering the dependence of tractography on the seeding location, the connectivity probability from *i* to *j* is not necessarily equivalent to that from *j* to *i*. Therefore, we computed the undirected connectivity probability *Nij* between regions *i* and *j* by averaging these two probabilities and defined it as the edge weight. To minimize possible false-positive fiber streamlines, edges with a weight value below 0.01 were considered potentially spurious and were removed from the network matrix, which was consistent with our previous research (17). The same threshold was applied to both patients and controls in order not to artificially inflate any group difference. As a result, an individual-specific 90 × 90 weighted network matrix was generated for each participant. Moreover, to define the rich-club topology, the unweighted brain networks were obtained as connections were represented in a binary fashion (connection present = 1, connection absent = 0). In detail, the edges with a connectivity probability above 0.01 were assigned a value of 1, and the edges with a connectivity probability below 0.01 were assigned a value of zero. Then, an individual-specific binary network matrix was generated for each participant. Next, for both patients and healthy controls, a group-averaged binary network was generated by selecting all the edges that were present in at least 75% of the subjects for each group. These steps were also reported in previous articles (16, 26) and were considered helpful for alleviating noise caused by inter-subject variability.

### 2.6. Graph analysis of connectome topology

#### 2.6.1. Global network metrics

To examine the possible differences in whole-brain network topology between patients and controls, several characteristic graph properties of the brain network of interest were computed using the Brain Connectivity Toolbox (http://sites.google.com/a/brain-connectivity-toolbox.net/bct/metrics) (27). All properties were calculated based on weighted networks to better describe the information of the existing connections. The global network topological properties were described in terms of the global connection strength, global efficiency, and small-worldness (details described in Supplementary material). Each metric provided a specific viewpoint to characterize the main features of the large-scale architecture.

#### 2.6.2. Rich-club organization

The focus of our study was the investigation of rich-club organization of the structural brain network in CAE patients. The so-called rich-club phenomenon in networks is said to be present when a small set of these highly connected hub regions tend to be more densely interconnected among each other than expected by chance (16). The rich club is thought to facilitate the integration of brain functions by providing shorter and faster routes of information transfer across widespread brain networks. In this study, the rich-club organization was delineated in the binary network. A detailed description of the unweighted rich-club coefficient is given in the Supplementary material. In short, for the given nodal degree *k* (the number of connections attached to each network node), the subnetwork comprising nodes with a degree larger than *k* was selected. For the remaining network, the rich-club coefficient Φ(*k*) was calculated as the ratio of connections present in the subnetwork and the total number of possible connections that would be present when all these remaining nodes would be fully connected. Then, the normalized rich-club coefficient Φ_norm_(*k*) was obtained by comparing and normalizing the rich-club coefficient to a set of 1000 comparable random networks with the same number of nodes, edges, and preserved degree distribution. By definition, a normalized rich-club coefficient Φ_norm_(*k*) >1 over a range of *k* is indicative of a rich-club organization in a network (16).

#### 2.6.3. Node and edge classification

Once a rich-club organization has been identified, network nodes can be classified as rich-club nodes and non-rich-club nodes, and existing connections in the network were classified into three categories: (1) rich-club connections only linking rich-club nodes among each other, (2) feeder connections linking rich-club to non-rich-club nodes, and (3) local connections only linking non-rich-club nodes among each other (**Figure 3C**). This classification was commonly adopted in several analyses and helpful for the estimation and statistical comparison of multiple aspects of brain connections (16, 26).

For each individual dataset, rich club, feeder, and local connection strength were computed as a sum of all the weights of their connections, respectively.

### 2.7. Statistical analysis

To evaluate the group differences in graphic metrics between patients and healthy controls, permutation testing (10000) was used for randomizing group assignments (28, 29). For rich-club analysis, a *p*-value was corrected for the number of rich-club levels.

In addition, to investigate how impairments in rich-club topology relate to clinical characteristics in CAE, we used Pearson's correlations to assess how rich-club connectivity related to disease duration and seizure frequency for patients.

## 3. Results

### 3.1. Global network metrics

**Figure 2:**
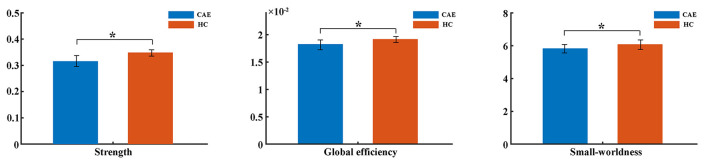
Comparisons of global network metrics between CAE patients and healthy controls (HC). The asterisk shows a significant between-group difference (^*^p < 0.05). The error bar indicates the standard deviation.

Consistent with our previous report on global network topology in CAE patients (17), this study further confirmed the topological alterations with a larger sample. The bivariate comparison showed that the strength and global efficiency of the structural network were significantly reduced in patients relative to healthy controls (*p* = 0.01 and *p* = 0.03, respectively) (Figure 2). Moreover, both groups got a small-world organization within the structural brain network as the value of σ>1, while the small-worldness was reduced in patients (*p* = 0.04) (Figure 2).

### 3.2. Rich-club organization

**Figure 3:**
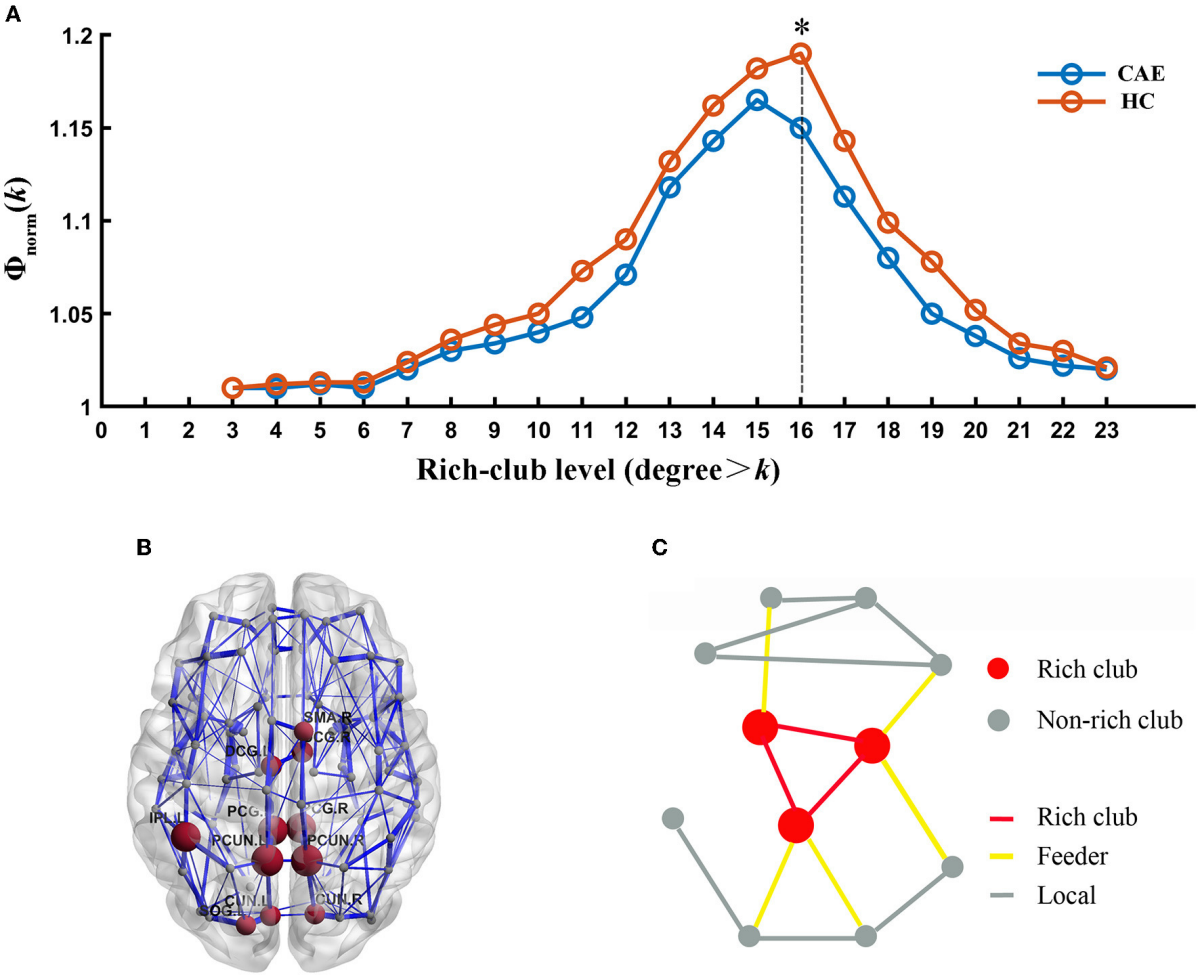
Rich-club organization. **(A)** Normalized rich-club coefficient (Φnorm) at different rich-club levels expressed as a nodal degree for patients and healthy controls. (*) Significantly reduced Φnorm in CAE subjects compared with healthy controls. **(B)** Network representation of rich-club regions (brown). **(C)** Schematic illustration of rich-club (red) and non-rich-club (gray) nodes and local (gray), feeder (yellow), and rich-club (red) connections.

Group averaged rich-club curves of both the patient and control networks are illustrated in Figure 3A. Based on the definition of Φ_norm_(*k*) > 1, both patients and controls had a rich-club organization in structural networks, but patients showed a reduced rich-club coefficient. Notably, the reduction in Φ_norm_ in patients was most pronounced at the level of k = 16 (*p* = 0.008). Rich-club regions were then selected based on this level, including the bilateral posterior cingulate gyrus, bilateral median cingulate and paracingulate gyri, bilateral precuneus, bilateral cuneus, left superior occipital gyrus, right supplementary motor area, and left inferior parietal gyrus (Figure 3B). This decision was in line with previous studies (16, 30).

**Figure 4:**
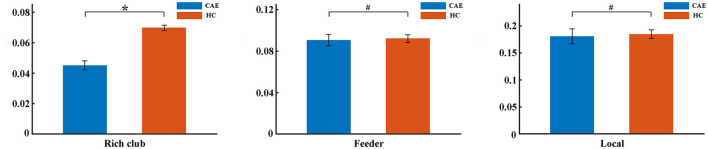
Differences of rich-club organization between patients and controls. The group differences in strength for rich-club connections, feeder connections, and local connections were displayed, respectively (^*^p < 0.05, ^#^*p* > 0.05). The error bar indicates the standard deviation.

Further analysis of the connections revealed a lower rich-club connectivity strength in patients (*p* = 0.002, Figure 4). In contrast, this alteration was not significant for feeder connections and local connections (*p* = 0.27 and *p* = 0.99, respectively, Figure 4).

**Figure 5:**
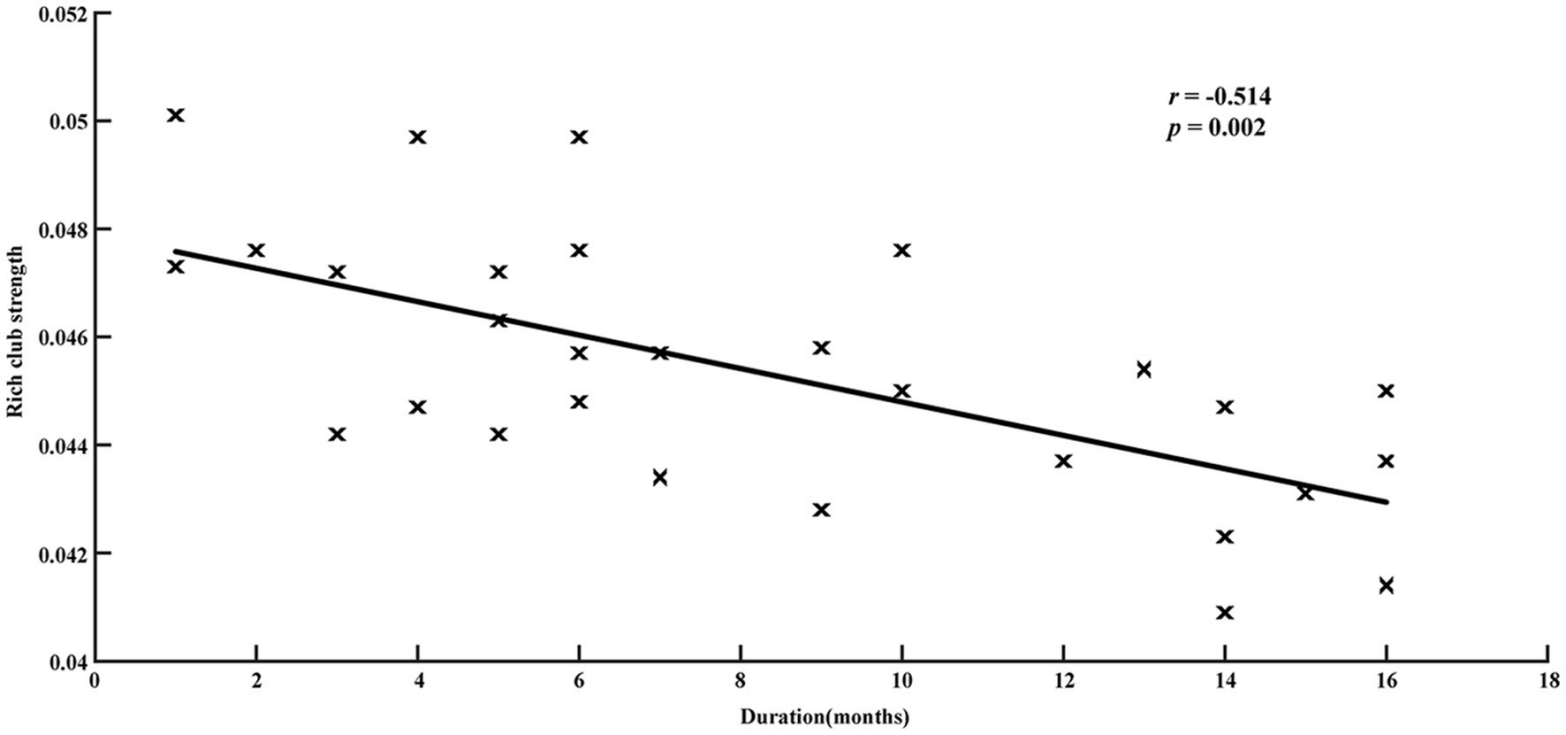
Relationship between disease duration and rich-club strength in patients group. Significantly negative correlations are revealed (*r* = −0.514, *p* = 0.002).

Correlation analysis showed that the reduction in rich-club strength was significantly related to disease duration (*r* = −0.514, *p* = 0.002, Figure 5), while no significant correlation was detected between the rich-club topology and seizure frequency (Supplementary Figure S1).

## 4. Discussion

This study investigated the structural network, especially the rich-club topology, in CAE using the DTI dataset and graph theory. Lower network strength and global efficiency were detected in this study, indicating a less efficient network in childhood absence epilepsy. The weakening shift of brain topology had been consistently reported in previous studies (17, 31). The small-world topology falls between regular and random networks and can support both segregated and integrated information processing (32). In this research, small-world topology was proven in both patients and controls following the criterion of small-worldness index (σ) > 1 (33). However, the index of σ decreased significantly in patients, which may correspond to a disruption of the balance between information integration and segregation.

The main focus of this study was the alteration of structural rich-club topology in clinical CAE patients. As referred, the densely connected and highly centralized hub brain regions had a strong tendency to be connected among themselves to form a “rich club,” which might serve as a connectivity backbone in the structural networks of the human brain (26). Although rich-club architecture in rodents and many human neuropsychiatric disorders have been extensively explored in recent years (34–38), it is still unclear in CAE. Our study was the first to explore the rich-club topology of the structural brain network in CAE using graph theory. Previous studies had provided new insights into the topological organization of structural connectome in CAE; however, they were limited to network-based analysis or global metrics and were not able to address the selective vulnerability of rich-club organizations.

In this study, we were able to show that the structural brain network of CAE derived from the DTI dataset by probabilistic tractography indeed got a rich-club organization, but the rich-club connectivity in patients with CAE was significantly reduced, reflecting a lower level of connectivity among hub brain regions. Immature rich-club topology might be related to some neurodevelopmental disorders (39). Recently, emerging evidence of brain network abnormalities has been reported in patients with CAE, such as impaired attention network (40) and salience network (41), altered effective connectivity (42), and disrupted structural connectivity in orbitofrontal and sub-cortical regions (31). Going beyond these findings, our study revealed an impaired rich-club organization while the feeder and local connections seem to be relatively reserved. This may be explained by the vulnerability of rich-club regions in brain disorders as these regions are characterized by not only high topological value but also high biological cost (14).

Furthermore, the association between the impaired rich-club organization and disease effects was found in this study. Lower rich-club connection strength was statistically correlated with longer disease duration of absence epilepsy, implying the longer the duration, the worse the impairment. This was in good agreement with previous reports concerning the long-standing burden of epileptic discharges on brain dysfunction (43–45). Although whether the structural abnormalities are reasons or consequences of frequent absence seizure still remains unclear, we tentatively speculate that impaired rich-club topology might be the consequence of long-term injurious effects of epileptic activity. As a result, an altered rich-club organization may serve as a potential biomarker for disease progression in CAE, which needs to be further discussed.

## 5. Limitations

Although we reported some promising findings in this study, there were also some issues that should be considered when interpreting these results. First, node definition is an important issue. The results may vary with the number of parcellated regions, region size, or their locations when using graph theory to analyze brain networks (46). It was strongly suggested that the high-resolution network keeps a great consistency with regional analysis in investigating the existence of a densely interconnected rich club in the brain connectome (16). However, replicating the current dataset in an alternative parcellation scheme is still deserved in future work to get a more convincing result. Second, given the correlation between rich-club alterations and disease duration, we supported the hypothesis that rich-club impairments might be the consequence of long-term frequent epileptic discharges, but this hypothesis might be preliminary due to the cross-sectional nature of our study and relatively small sample size (47). Therefore, future longitudinal studies with larger samples are advisable to further examine the causal relationship between epileptic activity and rich-club changes in this regard. Finally, it is the trouble of the network model. In addition to the binary network, there were also some other weighted networks, such as the streamline-weighted network and FA-weighted network, which had been widely discussed in previous studies (16, 35). Unfortunately, there has been no consensus on the selection of a network model in rich-club analysis.

## 6. Conclusion

Our study further confirmed the severely disrupted connectome in CAE with a larger sample as network strength, global efficiency, and small-worldness significantly decreased. More importantly, this study detected an abnormal rich-club organization in CAE, and these impairments were significantly correlated with disease duration. All these findings highlight the role of the rich club as a backbone for the brain connectome and provide a novel perspective to understand the pathophysiological mechanism of CAE.

## Data availability statement

The original contributions presented in the study are included in the article/Supplementary material, further inquiries can be directed to the corresponding author.

## Ethics statement

The studies involving human participants were reviewed and approved by the Ethics Committee of Nanjing Brain Hospital Affiliated to Nanjing Medical University and the Ethics Committee of Huai'an Hospital Affiliated to Xuzhou Medical University. Written informed consent to participate in this study was provided by the participants' legal guardian/next of kin.

## Author contributions

YY, WQ, XC, and YZ designed the research. MQ, YT, and JT analyzed the data. Participants were recruited and images were acquired by WZ and XC. The manuscript's textual content was written by YY and MQ and revised by WQ. All authors approved the final submitted version and agreed to be accountable for its content.
