# Supplementary material for: Impaired rich-club connectivity in childhood absence epilepsy
Source: Front Neurol. 2023 May 11;14:1135305. doi: 10.3389/fneur.2023.1135305 (PMC10213928; doi:10.3389/fneur.2023.1135305)
Supplement: Supplementary file 1 [file Table_1.DOCX]

| **Table S1 Demographic and clinical data of patients** | | | | | | |
| --- | --- | --- | --- | --- | --- | --- |
| subjects ID | sex | age  (y) | disease duration  (m) | frequency of seizure  (times/d) | ASMs | frequency of SWDs  (Hz) |
| 1 | M | 10 | 20 | 7 | VPA | 2-3 |
| 2 | M | 8 | 4 | 4 | VPA | 2.5-3 |
| 3 | M | 7 | 7 | 10 | None | 3 |
| 4 | F | 9 | 9 | 6 | LEV | 3 |
| 5 | M | 8 | 5 | 16 | VPA | 3-3.5 |
| 6 | F | 7 | 5 | 6 | VPA | 3 |
| 7 | F | 9 | 6 | 1 | None | 2-3 |
| 8 | M | 6 | 4 | 5 | VPA | 3 |
| 9 | F | 7 | 4 | 14 | VPA | 3 |
| 10 | F | 9 | 10 | 3 | LEV | 3 |
| 11 | M | 5 | 5 | 7 | VPA | 3 |
| 12 | F | 8 | 18 | 3 | None | 3-3.2 |
| 13 | M | 10 | 9 | 5 | LEV | 3 |
| 14 | M | 11 | 15 | 16 | VPA, LEV | 3 |
| 15 | M | 8 | 10 | 4 | VPA | 2.5-3 |
| 16 | F | 9 | 11 | 4 | LEV | 3 |
| 17 | M | 9 | 11 | 6 | VPA | 2.5-3.5 |
| 18 | F | 6 | 2 | 13 | LEV | 3 |
| 19 | F | 11 | 20 | 9 | None | 2.5-3.5 |
| 20 | F | 10 | 2 | 15 | LEV | 3 |
| 21 | F | 6 | 3 | 9 | VPA | 3 |
| 22 | M | 8 | 1 | 1 | VPA, LEV | 2.5-3 |
| 23 | F | 8 | 3 | 2 | None | 3 |
| 24 | M | 10 | 7 | 9 | VPA | 3 |
| 25 | F | 7 | 11 | 2 | LEV | 2.5-3.5 |
| 26 | M | 9 | 12 | 18 | VPA | 2.5-3.5 |
| 27 | F | 7 | 13 | 21 | VPA, LEV | 3 |
| 28 | F | 11 | 14 | 17 | VPA, LEV | 3 |
| 29 | M | 10 | 18 | 8 | VPA | 3 |
| 30 | F | 6 | 21 | 7 | LEV | 2-3 |
| M=male; F=female; d=day; m=months; y=years; ASMs=anti-seizure medications; LEV=levetiracetam; VPA=valproic acid; SWDs = spike wave discharges. | | | | | | |
