# Supplementary material for: Impaired rich-club connectivity in childhood absence epilepsy
Source: Front Neurol. 2023 May 11;14:1135305. doi: 10.3389/fneur.2023.1135305 (PMC10213928; doi:10.3389/fneur.2023.1135305)
Supplement: Supplementary file 2 [file Table_2.docx]

**Table S2** Regions of interest (45 in each cerebral hemisphere) defined in this study using AAL atlas.

| **Index** | **Regions** | **Abbreviation** |
| --- | --- | --- |
| 1 | Precentral | PreCG |
| 2 | Superior frontal gyrus (dorsolateral) | SFGdor |
| 3 | Superior frontal gyrus (orbital part) | ORBsup |
| 4 | Middle frontal gyrus | MFG |
| 5 | Middle frontal gyrus (orbital part) | ORBmid |
| 6 | Inferior frontal gyrus (opercular part) | IFGoperc |
| 7 | Inferior frontal gyrus (triangular part) | IFGtriang |
| 8 | Inferior frontal gyrus (orbital part) | ORBinf |
| 9 | Rolandic operculum | ROL |
| 10 | Supplementary motor area | SMA |
| 11 | Olfactroy cortex | OLF |
| 12 | Superior frontal gyrus (medial) | SFGmed |
| 13 | Superior frontal gyrus (medial orbital) | ORBsupmed |
| 14 | Rectus gyrus | REC |
| 15 | Insula | INS |
| 16 | Anterior cingulate gyri | ACG |
| 17 | Median cingulate gyri | MCG |
| 18 | Posterior cingulate gyrus | PCG |
| 19 | Hippocampus | HIP |
| 20 | Parahippocampalgyrus | PHG |
| 21 | Amygdala | AMYG |
| 22 | Calcarine fissure | CAL |
| 23 | Cuneus | CUN |
| 24 | Lingual gyrus | LING |
| 25 | Superior occipital gyrus | SOG |
| 26 | Middle occipital gyrus | MOG |
| 27 | Inferior occipital gyrus | IOG |
| 28 | Fusiform gyrus | FFG |
| 29 | Postcentralgyrus | PoCG |
| 30 | Superior parietal gyrus | SPG |
| 31 | Inferior parietal gyrus | IPG |
| 32 | Supramarginalgyrus | SMG |
| 33 | Angular gyrus | ANG |
| 34 | Precuneus | PCUN |
| 35 | Paracentral lobule | PCL |
| 36 | Caudate nucleus | CAU |
| 37 | Putamen | PUT |
| 38 | Pallidum | PAL |
| 39 | Thalamus | THA |
| 40 | Heschlgyrus | HES |
| 41 | Superior temporal gyrus | STG |
| 42 | Superior temporal gyrus, temporal pole | TPOsup |
| 43 | Middle temporal gyrus | MTG |
| 44 | Middle temporal gyrus, temporal pole | TPOmid |
| 45 | Inferior temporal gyrus | ITG |

The abbreviations are listed according to Salvador [[1](#_ENREF_1)], which differ slightly from the original abbreviations by Tzourio-Mazoyer [[2](#_ENREF_2)].

1. Salvador R, Suckling J, Coleman MR, Pickard JD, Menon D, et al. (2005) Neurophysiological architecture of functional magnetic resonance images of human brain. Cereb Cortex 15: 1332-1342.

2. Tzourio-Mazoyer N, Landeau B, Papathanassiou D, Crivello F, Etard O, et al. (2002) Automated anatomical labeling of activations in SPM using a macroscopic anatomical parcellation of the MNI MRI single-subject brain. Neuroimage 15: 273-289.
