# Supplementary material for: Impaired rich-club connectivity in childhood absence epilepsy
Source: Front Neurol. 2023 May 11;14:1135305. doi: 10.3389/fneur.2023.1135305 (PMC10213928; doi:10.3389/fneur.2023.1135305)
Supplement: Supplementary file 3 [file Data_Sheet_1.docx]

Supplementary Material

Impaired rich-club connectivity in childhood absence epilepsy

**Yadong Yu^1†^, Mengdi Qiu^2†^, Wenwei Zou^3^, Ying Zhao^3^, Yan Tang^3^, Jisha Tian^3^, Xiaoyu Chen^4^, Wenchao Qiu^3*^**

**^1^ Department of Neurology, Lianshui County People’s Hospital, Huai’an, China**

**^2^ Department of Neurology, The Fifth People’s Hospital of Huai’an, Huai’an, China**

**^3^ Department of Neurology, The Affiliated Huai’an Hospital of Xuzhou Medical University, Huai’an, China**

**^4^ Department of Radiology, The Affiliated Huai’an Hospital of Xuzhou Medical University, Huai’an, China**

*** Correspondence:**

**Wenchao Qiu**

**wenchaoqiu2010@163.com**

^†^ **These authors contributed equally to this work and share first authorship**

# Supplementary Data

**Strength**

Strength of node ***i*** is defined as the sum of edge weights linking to it [[1](#_ENREF_1)], while network strength means the average of the strengths across all the nodes.

$$S(G)=\frac{1}{N}\sum_{i\in G} S_{i}$$

Where ***N*** is the number of nodes in graph (network) ***G*** and ***S_i_*** is the sum of the edge weights ***w_ij_*** connecting to node ***i***.

**Efficiency**

Efficiency of network, including global and local efficiency, is a measure of how efficiently it exchanges information in the network [[2](#_ENREF_2)]. The global efficiency (***E_glob_***) represents the capability of information flow over the entire network. In this work, we defined the global efficiency using Dijkstra's algorithm [[3](#_ENREF_3),[4](#_ENREF_4)].

$$E_{glob}(G)=\frac{1}{N(N-1)}\sum_{i\neq j\in G} \frac{1}{L_{ij}}$$

Where ***L_ij_*** is the shortest path length between node ***i*** and node ***j*** in ***G***.

**Small-worldness**

Small-world measures, including clustering coefficient (***C_P_***), characteristic path length (***L_P_***), normalized clustering coefficient (***γ***), normalized characteristic path length (***λ***), and small-worldness (***σ***), are most frequently used properties in brain network study [[5](#_ENREF_5)]. Highly interconnected neighbors around a given node form a cluster, while sparsely interconnected neighbors do not. Clustering coefficient of node ***i*** (***C_i_***) reflects the number of connections among the neighbors of node ***i***.

$$C_{i}=\frac{2t_{i}}{k_{i}(k_{i}-1)}$$

Where ***k_i_*** is the degree of node ***i*** and ***t_i_*** is the number of triangles around node ***i***.

Moreover, the clustering coefficient of a network (***C_P_***) is quantified as the average of the clustering coefficient over all nodes, which characterizes network segregation.

$$C_{P}(G)=\frac{1}{N}\sum_{i\in G} C_{i}$$

Characteristic path length (***L_P_***) is defined as the average of shortest path length between all nodal pairs, which characterizes the integration or information transfer capacity across remote cortical regions.

$$L_{P}=\frac{1}{N(N-1)}\sum_{i\neq j\in G} L_{ij}$$

Where ***L_ij_*** is the shortest path length from node ***i*** to node ***j***.

Moreover, the normalized clustering coefficient (***γ***) and the normalized characteristic path length (***λ***) were obtained from comparing ***C_P_*** and ***L_P_*** of brain network with that of 100 random networks with the same number of nodes and degree distribution.

$$\gamma=\frac{C_{P}}{C_{P}^{rand}}, \lambda=\frac{L_{P}}{L_{P}^{rand}}$$

Where $\boldsymbol{C}_{\boldsymbol{P}}^{\boldsymbol{rand}}$ and $\boldsymbol{L}_{\boldsymbol{P}}^{\boldsymbol{rand}}$ are the mean of ***C_P_*** and ***L_P_*** of 100 matched random networks.

The brain network would be considered as small-world if $\gamma\gg1$ and$\lambda\approx1$. Furthermore, these two properties can be summarized into a simple quantitative metric, small-worldness ($\boldsymbol{\sigma}$):

$$\sigma=\frac{\gamma}{\lambda}$$

which is higher than 1 for the small-world network [[6](#_ENREF_6)].

**Rich club coefficient**

For a given network, the degree of each node ***i*** was determined as ***ki***. All nodes that showed a number of connections of ≤ ***k*** were removed from the network. For the remaining network, the rich-club coefficient **Φ**(*k*) was computed as the ratio of connections present between the remaining nodes and the total number of possible connections that would be present when the set would be fully connected[[7](#_ENREF_7)].

$$\text{Φ(k)=}\frac{{2E}_{>k}}{N_{>k}(N_{>k}-1)}$$

Due to the fact that nodes with a higher degree also have a higher probability of being interconnected by chance alone, Φ(*k*) is typically normalized relative to a set of comparable random networks of equal size and similar connectivity distribution, giving a normalized rich-club coefficient Φ_norm_[[8](#_ENREF_8),[9](#_ENREF_9)]. In this study, for each network, *m*=1000 random networks were computed, keeping the degree distribution and sequence of the matrix intact[[10](#_ENREF_10)], and from each of the randomized networks, the rich-club coefficient Φ_random_ was computed for each level of *k*. Next, the overall Φ_random_(*k*) was computed as the average rich-club coefficient over the *m* random networks. The normalized rich-club coefficient Φ_norm_(*k*) was computed as follows:

$$\Phi_{norm}\left( k \right)=\frac{\Phi(k)}{\Phi_{random}(k)}$$

An increasing normalized coefficient Φ_norm_ of >1 over a range of *k* reflects the existence of rich-club organization in a network[[7](#_ENREF_7)]. For simplicity, Φ_norm_(*k*) will be referred to as Φ(*k*) in the main text.

**References**

1. Barrat A, Barthelemy M, Pastor-Satorras R, Vespignani A (2004) The architecture of complex weighted networks. Proc Natl Acad Sci U S A 101: 3747-3752.

2. Achard S, Bullmore E (2007) Efficiency and cost of economical brain functional networks. PLoS Comput Biol 3: e17.

3. Onnela JP, Saramaki J, Kertesz J, Kaski K (2005) Intensity and coherence of motifs in weighted complex networks. Phys Rev E Stat Nonlin Soft Matter Phys 71: 065103.

4. Latora V, Marchiori M (2001) Efficient behavior of small-world networks. Phys Rev Lett 87: 198701.

5. Watts DJ, Strogatz SH (1998) Collective dynamics of 'small-world' networks. Nature 393: 440-442.

6. Humphries MD, Gurney K (2008) Network 'small-world-ness': a quantitative method for determining canonical network equivalence. PLoS One 3: e0002051.

7. van den Heuvel MP, Sporns O (2011) Rich-club organization of the human connectome. J Neurosci 31: 15775-15786.

8. McAuley J, da F. Costa L, Caetano T (2007) Rich-club phenomena across complex network hierachies. Applied Physics Letters 91.

9. Colizza V, Flammini A, Serrano M, Vespignani A (2006) Detecting rich-club ordering in complex networks. Nat Phys 2.

10. Rubinov M, Sporns O (2010) Complex network measures of brain connectivity: uses and interpretations. Neuroimage 52: 1059-1069.

# Supplementary Figures and Tables

Figure legend

Figure S1 Scatter plot of seizure frequency and rich club strength in patients group. No significantly correlation was revealed.
