# Supplementary figures and images for: Impaired rich-club connectivity in childhood absence epilepsy
Source: Front Neurol. 2023 May 11;14:1135305. doi: 10.3389/fneur.2023.1135305 (PMC10213928; doi:10.3389/fneur.2023.1135305)

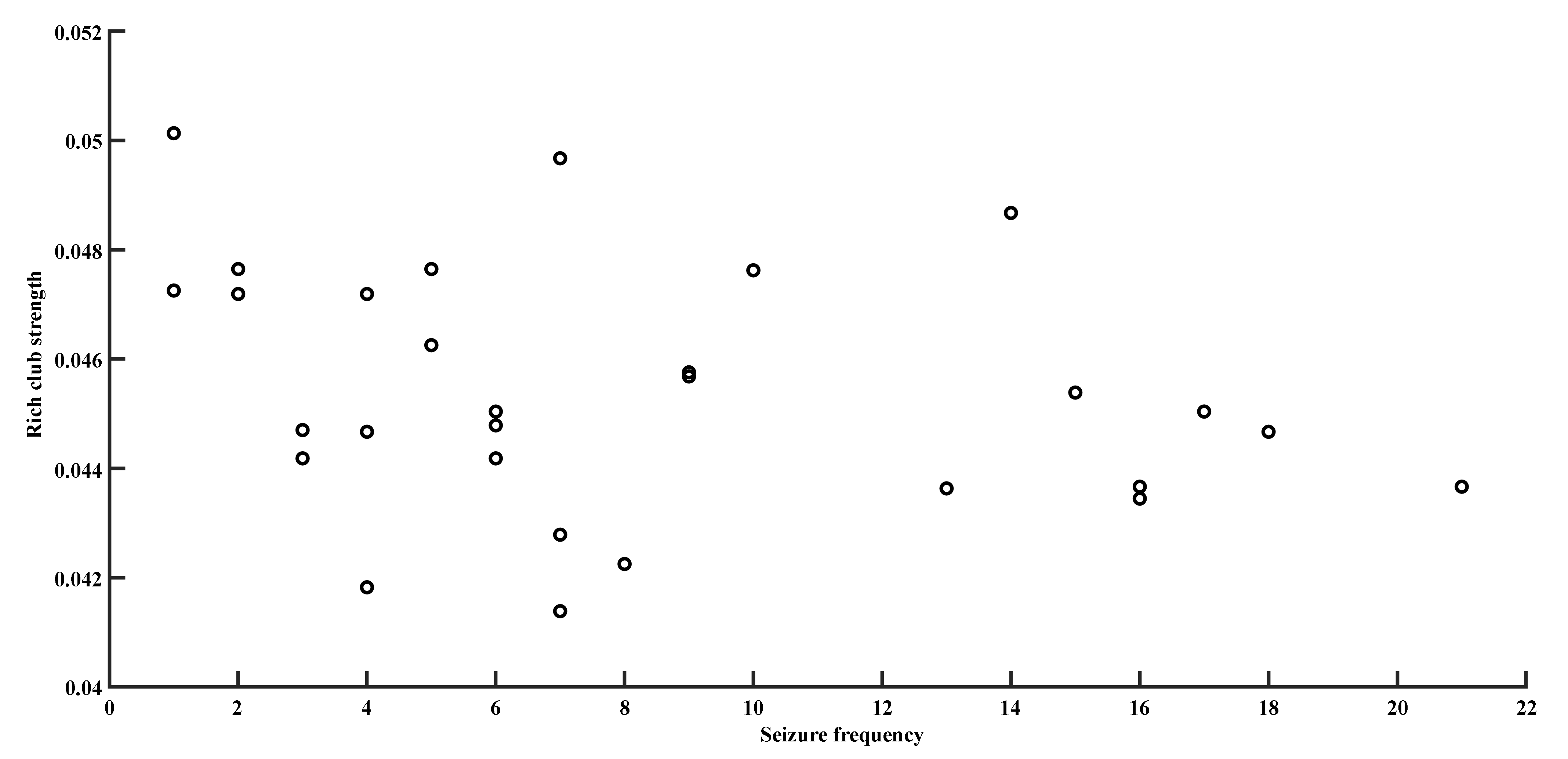

Supplement: Supplementary file 4 [file Image_1.TIF]
